# Biomechanical investigation of extragraft bone formation influences on the operated motion segment after anterior cervical spinal discectomy and fusion

**DOI:** 10.1038/s41598-019-54785-9

**Published:** 2019-12-11

**Authors:** Won Man Park, Yong Jun Jin

**Affiliations:** 1 Elsoltec Inc., Yongin, Korea; 2 Department of Neurosurgery, Inje University College of Medicine, Seoul Paik Hospital, Seoul, Korea

**Keywords:** Computational models, Biomedical engineering

## Abstract

Although the clinical importance of extragraft bone formation (ExGBF) and bridging (ExGBB) has been reported, few studies have investigated the biomechanical influences of ExGBF on the motion segment. In this study, ExGBF was simulated at the C5-C6 motion segment after anterior cervical discectomy and fusion using a developed finite element model and a sequential bone-remodelling algorithm in flexion and extension. The computer simulation results showed that extragraft bone was primarily formed in the extension motion and grew to form ExGBB. A stepwise decrease in the intersegmental rotation angle, maximum von Mises stress and strain energy density on the trabecular bone with ExGBF were predicted in extension. When ExGBB was formed in the trabecular bone region, the intersegmental rotation angle slightly decreased with additional bone formation. However, the stress and strain energy density on the trabecular bone region decreased until ExGBB reached the peripheral cortical margin. The results offer a rationale supporting the hypothesis that mechanical stimuli influence ExGBF. ExGBF was helpful in increasing the stability of the motion segment and decreasing the fracture risk of trabecular bones, even in cases in which ExGBB was not formed. ExGBB can be classified as either soft or hard bridging based on a biomechanical point of view.

## Introduction

Since the 1950s, when the anterior cervical discectomy and fusion (ACDF) surgical method was first reported by Cloward^1^ and Robinson and Smith^2^, it has been widely used to treat patients with degenerated discs of the cervical spine. Autologous bone grafts have been applied for decades in ACDF, but patient dissatisfaction at the harvest site remains a weakness of the bone graft approach^3^. Currently, interbody cages are commonly used in ACDF as a safe alternative to iliac bone graft^4,5^, and they are generally used with allografts.

Achieving a solid interbody union with bridging trabeculae is critical for successful outcomes after ACDF. Allografts or bone substitutes filled in an interbody cage to lead intragraft bone bridging are used to achieve a solid interbody union. The formation of extragraft bone bridging (ExGBB) and intragraft bone bridging (InGBB), diagnosed using CT scans, are used as diagnostic criteria for ACDF. However, Song *et al*. reported that InGBB had the lowest specificity (50.9%) and a positive predictive value (34.3%), demonstrating a high false-positive rate for diagnosing solid fusion, whereas ExGBB had 100% sensitivity and a negative predictive value^5^. Therefore, the authors claimed that ExGBB is far more reliable and accurate compared with InGBB in determining anterior cervical fusion.

Bone formation around the inserted spinal implantation, not only with spinal cages but also with artificial discs, is common. However, it has not been clearly reported how bone is generated and grown. Lee *et al*., on the basis of a clinical study, revealed that ExGBB encroached the spinal canal in 21/27 (78%) patients after ACDF with a stand-alone cage and 6/31 (19%) patients after ACDF with an autologous iliac bone and plate fixation^6^. Both the difference in graft material and the addition of an anterior cervical plate were influential in the formation of ExGBB. This result indicates that the motion factor, which can be limited by an anterior cervical plate, may have a relationship with the overgrowth of ExGBB. In cases where a total disc was used, bone formation has also been reported in clinical studies^7–9^; Jin *et al*. reported that different types of bone formation occur according to the site on which different types of loading is applied^7^. Therefore, mechanical stimuli may be a major factor of bone formation around the inserted implant, but numerous factors, such as the male sex, an older age, multisegmental operations, bone dust, wear debris of the metal on the polyethylene component, and stress at the interface between the endplate and prosthesis, have been presumed to be causes of pathologic bone formation^10–12^.

Various computational simulation studies on the bone adaptation process based on strain energy density (SED) have shown that the simulated bone formation and resorption results are coincident with anatomical observations^13–19^ since Wolff introduced Wolff's law^20^. Weinans *et al*. predicted the distribution of bone mineral density at a proximal femur^13^, and Jang and Kim^14^, Boyle and Kim^15^, and Tsubota *et al*.^16^ simulated the detailed structure of the same component. Espinha *et al*. showed changes in the bone mineral density of the trabecular bone in vertebral bodies after ACDF^17^. Ganbat *et al*. investigated HO formation after total disc replacement (TDR), and these studies were the first studies on HO formation using the bone adaptation process^18,19^. However, the ExGBF after ACDF has not been simulated, and its biomechanical effects on the operated spine have not been studied. More specifically, biomechanical studies on the sequential growth of ExGBFs have not been performed.

In this study, we hypothesized that mechanical loading on the spinal MSU after cage implantation is related to ExGBF^7^. A sequential bone remodelling algorithm based on SED was developed and used with a three-dimensional finite element (FE) model of the cervical spine to simulate ExGBF. ExGBF during flexion and extension motions was investigated, and the changes in biomechanical behaviours were also analysed, including intersegmental rotation, stress and SED on the trabecular bone.

## Materials and Methods

### Development of an FE model of the intact cervical spine

**Figure 1:**
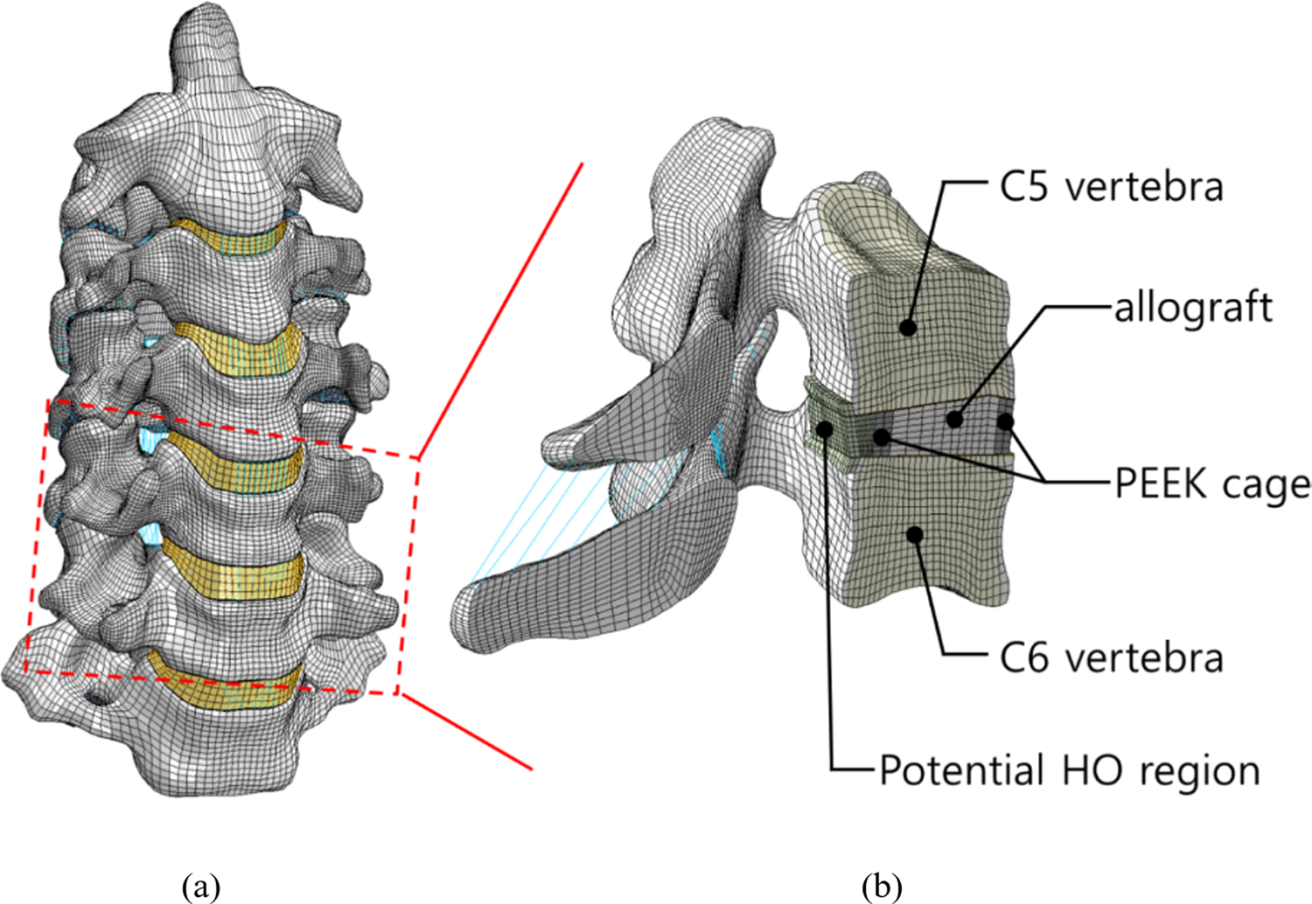
Developed FE models of the cervical spine: (**a**) intact spine from C2 to C7 and (**b**) operated C5-C6 motion segment with the ExGBF potential region.

A three-dimensional FE model of the C2-C7 spine, symmetric across the mid-sagittal plane, was developed using computed tomography (CT) images. The scans, with a thickness of 1 mm and resolution of 512 × 512 pixels, were obtained from a human database (cadaver, female, 69 years old, 157 cm, 55 kg, cause of death was pneumonia). Previously published modelling techniques and material properties were adapted (Fig. 1(a))^21–25^, but uncovertebral joints were not considered in this study. The model consisted of two spinal bones (consisting of cortical, cancellous, and post bones), end-plates (cartilaginous and bony endplates), six major ligaments (anterior longitudinal, posterior longitudinal, interspinal, supraspinal, flaval, and capsular ligaments), intervertebral discs (nucleus pulposus, annuls ground substance, and annuls fibrosus), and articular cartilages. IA-FEMesh^26^ and Abaqus/CAE 2018 (Simulia, Providence, RI, USA) were used to develop the FE model. The developed model was validated by comparing the intersegmental rotation at each MSU under pure bending moments of 1.0 Nm to previously published experimental results^27^.

### Development of an FE model of the operated motion segment unit

The C5-C6 MSU of the developed model was chosen to simulate ACDF. The anterior and posterior longitudinal ligaments were removed. The annulus fibrosus was partially removed for the discectomy. A PEEK standalone cage, (E = 4,000 MPa) created using the geometry of a PEEK cage (Solis cervical cage, Stryker), was inserted (Fig. 1(b)) after removal of the cartilaginous endplate. An allograft, assumed to be the trabecular bone, was filled in the cage. The anterior margin of the cage was aligned with the anterior margin of the vertebrae. The geometry of the stand-alone cage was simplified as follows: (1) the teeth of the cage were neglected, and a three-dimensional surface-to-surface contact condition with a high friction coefficient of 0.8 was applied on the contact surface between the vertebrae and the inserted cage^28^; (2) the ‘no separation option’, which prevents separation of the contact surface between the vertebrae and the cage, was applied to fix the implants on the vertebrae instead of modelling keels or screws.

### Modelling the potential region of ExGBF

The potential ExGBF region was predefined^18,19^ in the region posterior to the PEEK cage because bone formation occurs primarily in this region (Fig. 1(b)). The initial Young’s modulus (E) of the elements in the potential bone formation region was set to 1,474 MPa, which corresponds to a density (*ρ*) of 0.73 g/cm^3^, half of the maximum value of bone density. The formulation of Young’s modulus,

1 $${\rm{E}}=3790\,{\rho }^{3}$$

which was introduced in previously published studies, was used^13,29^. The maximum density was set to 1.47 g/cm^3^, which corresponds to a Young’s modulus of 12,000 MPa for the cortical bone. The potential ExGBF region was attached to the bony endplate and vertebrae, and a three-dimensional surface-to-surface contact condition with a friction coefficient of 0.8 was applied between the generated ExGBF and the inserted cage.

### Simulation of ExGBF

**Figure 2:**
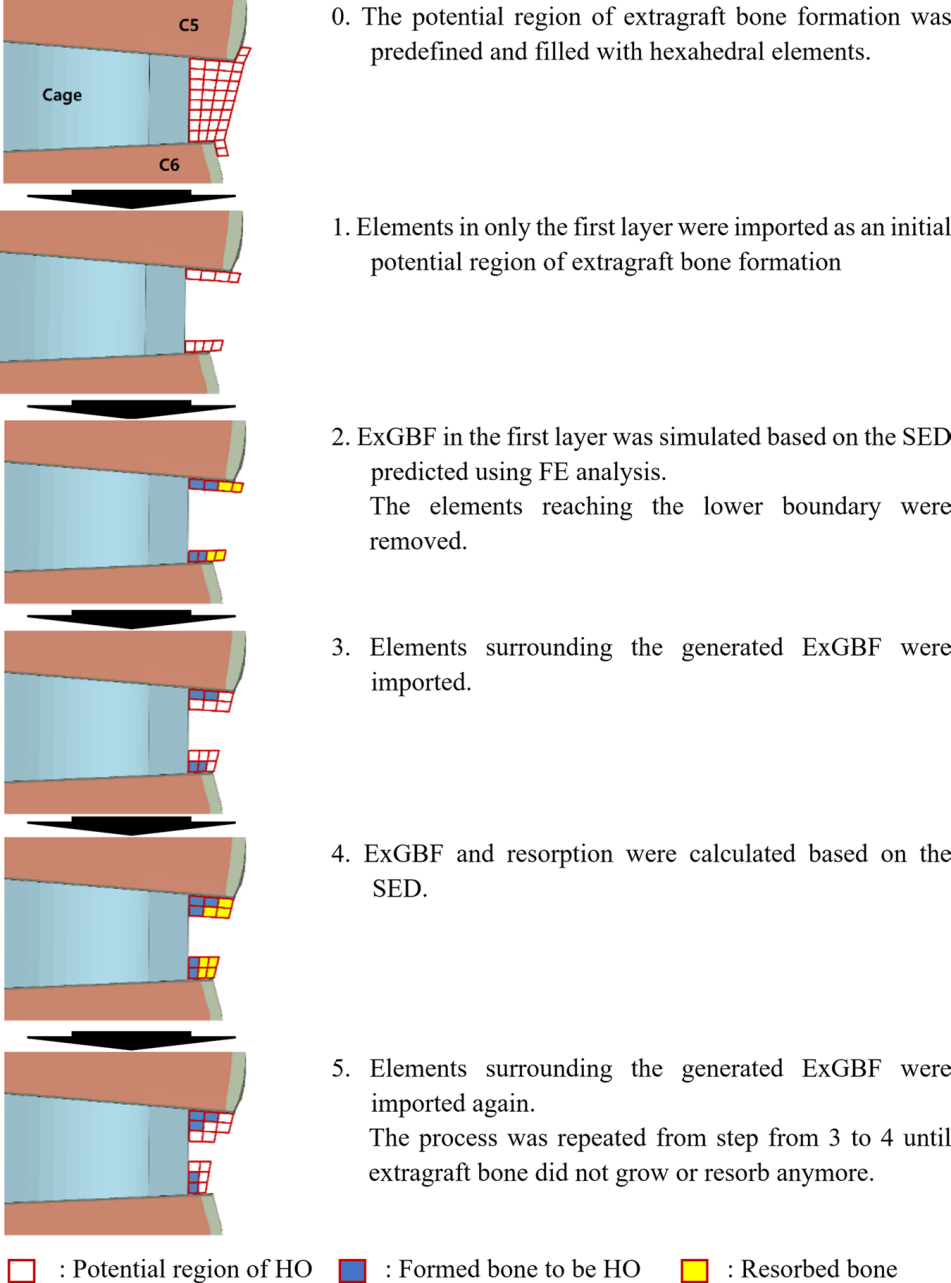
Schematic diagram of the sequential bone remodelling algorithm used to simulate ExGBF.

Custom MATLAB (MATLAB 2018a, MathWorks, Inc., Natick, MA, USA) code was developed to simulate sequential ExGBFs (Fig. 2). Initially, the layers of the potential ExGBF region adjacent to the upper and lower vertebrae were imported. The bone density *ρ* of each imported element was subsequently calculated as follows^13,19,30^,

2 $$\frac{d\rho }{dt}=\{\begin{array}{cc}B(S-(1+s)K), & S > (1+s)K\\ 0, & (1-s)K\le S\le (1+s)K\\ B(S-(1-s)K), & S < (1-s)K\end{array}$$

where unit time dt = 30, the remodelling rate coefficient B = 1.0 (g/cm^3^)^2^ and the SED per unit mass *S* = *U*/*ρ*. The homeostatic equilibrium SED K and the threshold range of the lazy zone s were chosen as 0.0131 J/g and 0.2443, respectively^31^. The Young’s modulus of each element in the potential ExGBF region was calculated using Eq. 1. The upper and lower boundaries of the Young’s modulus were set to 12,000 MPa and 0.1 MPa, respectively.

When the iteration of the bone adaptation process for the first layer was finished, the resorbed elements (in which the Young’s modulus reached the lower boundary) were removed, and the elements surrounding the formed elements were imported into the potential ExGBF region. The bone adaptation processes were performed again for both the remaining and newly imported elements. These processes were repeated until no changes occurred in the Young’s modulus of the elements for ExGBF.

ExGBFs were simulated in flexion and extension. A compressive force of 50 N was applied along the follower load direction. Flexion and extension moments of 1.5 Nm were applied on the superior plane of the C5 vertebra. The changes in the biomechanical behaviours of the operated MSU, including intersegmental rotation, stress, and SED, on the trabecular bone due to ExGBF were investigated and compared with those from the intact and operated FE models without ExGBF.

### Ethical approval and informed consent

In this study, computed tomography (CT) images collected from the Catholic Digital Human Library (Approval No. CUMC10U161, IRB, College of Medicine, The Catholic University of Korea) were used. Anonymized data, where personal information was concealed in such a way that the researcher could not identify the person, was used. In addition, informed consent was not required for this study because anonymized data of the cadavers were used.

## Results

### Validation of the developed FE model

**Figure 3:**
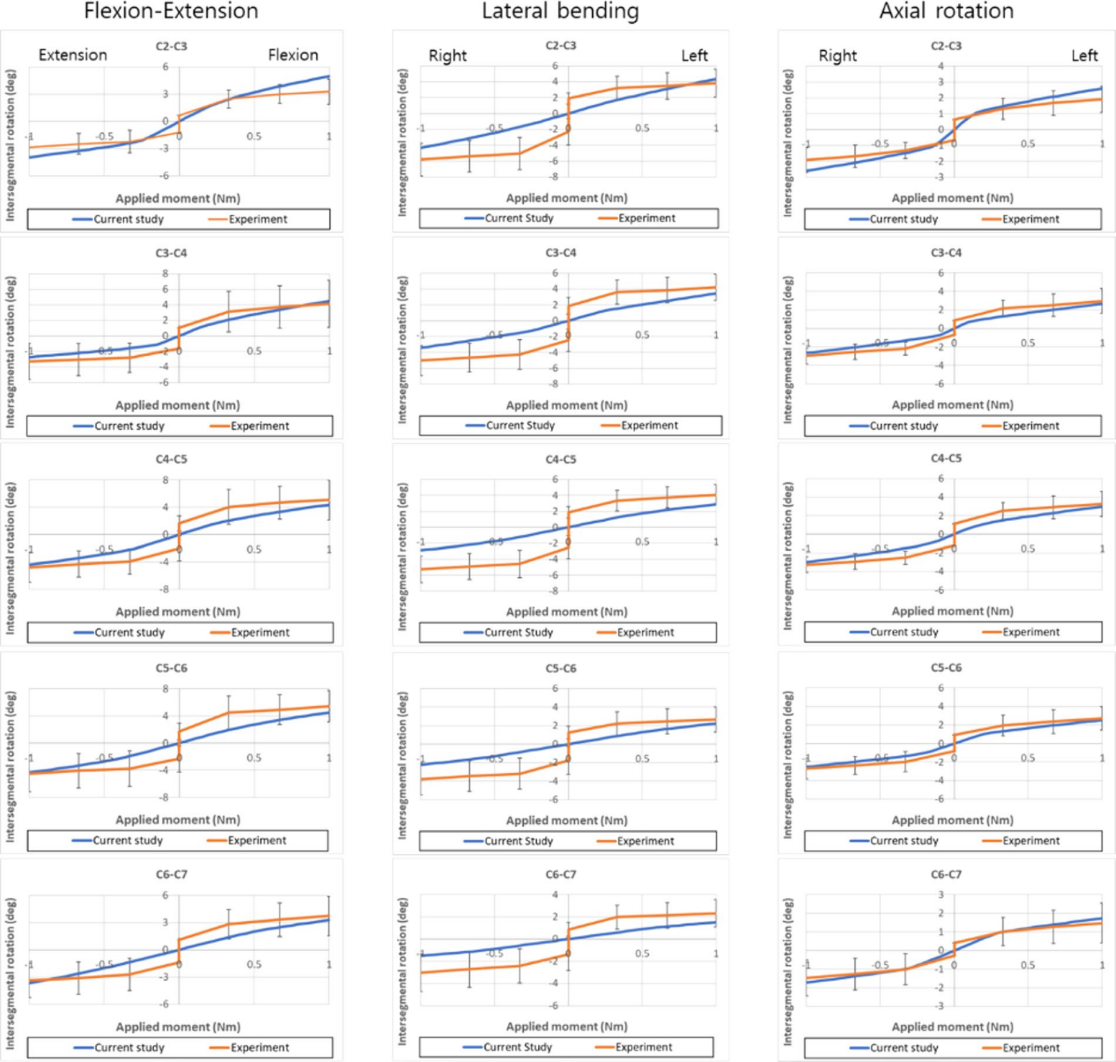
Moment-rotation curves of the developed FE model of the intact cervical spine predicted in pure bending moments along the flexion, extension, lateral bending, and axial rotation directions.

Moment-rotation curves along the flexion-extension, left-right lateral bending, and left-right axial rotation directions at each MSU were predicted in the developed FE model, and the curves were compared with those reported in the experimental study (Fig. 3)^27^. While the neutral zone, in which sudden changes in rotation occur around the neutral position, is shown in the results of the *in vitro* experiment, it was not shown in the results of FE analysis. Instead, relatively lower stiffness was shown in the neutral position than in the other positions in the analytical results. The developed model was symmetric across the mid-sagittal plane, and thus, the left lateral bending motions and axial rotations were the same as those to the right. The flexion, extension, left lateral bending, and left axial rotation angles under a moment of 1 Nm were 5.0°, 4.0°, 4.3° and 2.6° at C2-C3; 4.5°, 2.7°, 3.4° and 2.6° at C3-C4; 4.4°, 4.4°, 2.9° and 3.0° at C4-C5; 4.5°, 4.4°, 2.2° and 2.5° at C5-C6; and 3.3°, 3.7°, 1.5° and 1.7° at C6-C7, respectively. In the flexion-extension motion, only the predicted extension motion at C2-C3 exceeded the range in the experimental results under a moment of 1 Nm, but the other motions were within the range in the experimental results. The difference was only 0.40°. In the lateral bending motion, only the predicted motion at C4-C5 exceeded the range in the experimental results under a moment of 1 Nm. The differences were 0.85° and 0.08° in the right and left lateral bending, respectively. In axial rotation, all predicted motions were within the range of the experimental results.

### Simulation of ExGBF

**Figure 4:**
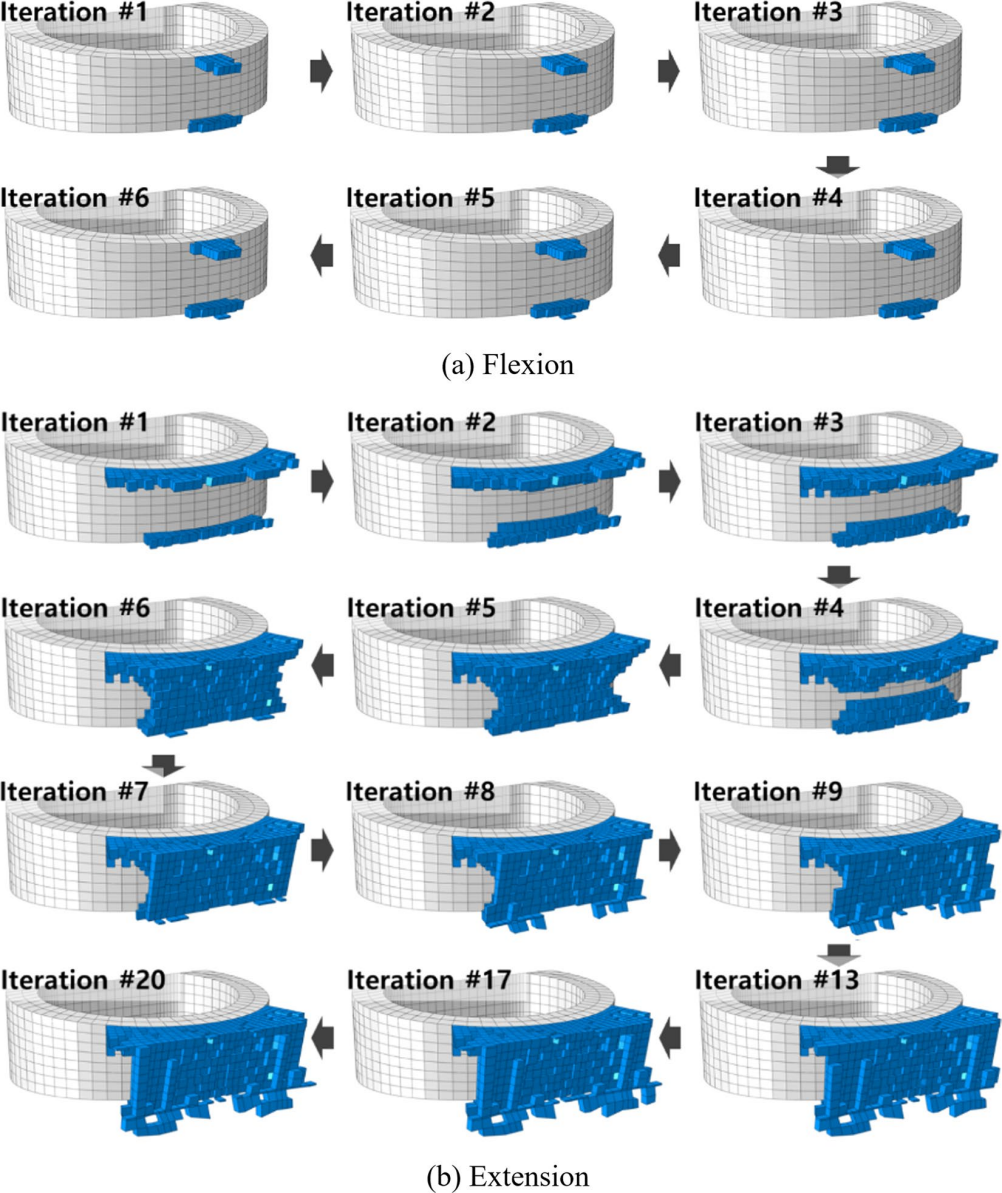
Sequential ExGBF during flexion and extension: (**a**) flexion and (**b**) extension.

**Figure 5:**
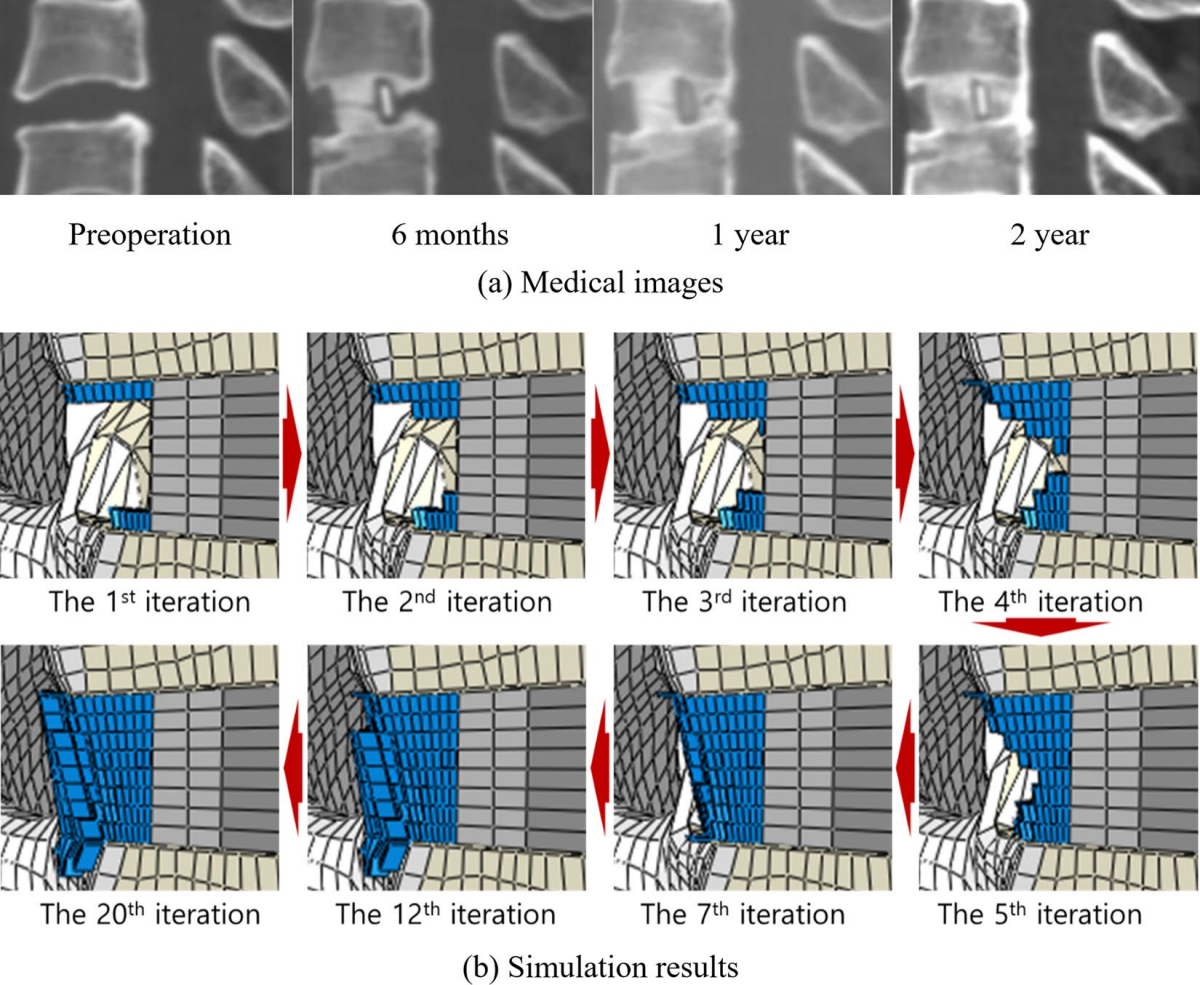
Comparison of ExGBF between (**a**) the medical images and (**b**) the simulation results.

The extension motion mainly influenced the ExGBF in the posterior region (Figs. 4 and 5). The initial formation was predicted to be at the posterior region of the cage adjacent to the vertebrae. ExGBFs grew towards each other until ExGBB was formed, and they grew towards the peripheral cortical margin in extension but did not grow in flexion. ExGBFs in extension were processed for 20 iterations. ExGBB in the trabecular bone region appeared at the 5th iteration and reached the peripheral cortical margin at the 10th iteration. The final volume of the ExGBF was 60.4 mm^3^, as predicted after the 20^th^ iteration (Table 1). Bone formation was terminated after 6 iterations in flexion, and the final volume was only 3.6 mm^3^.

**Table 1:** Volume of ExGBF in flexion and extension.

Flexion		Extension			
Iteration	Volume (mm^3^)	Iteration	Volume (mm^3^)	Iteration	Volume (mm^3^)
1	2.6	1	13.9	11	57.5
2	3.1	2	19.4	12	58.6
3	3.5	3	22.3	13	59.0
4	3.5	4	28.8	14	59.2
5	3.6	5	40.9	15	59.3
6	3.6	6	46.3	16	59.5
—	—	7	49.0	17	59.9
—	—	8	51.1	18	60.0
—	—	9	52.5	19	60.2
—	—	10	55.0	20	60.4

### Intersegmental rotation

**Figure 6:**
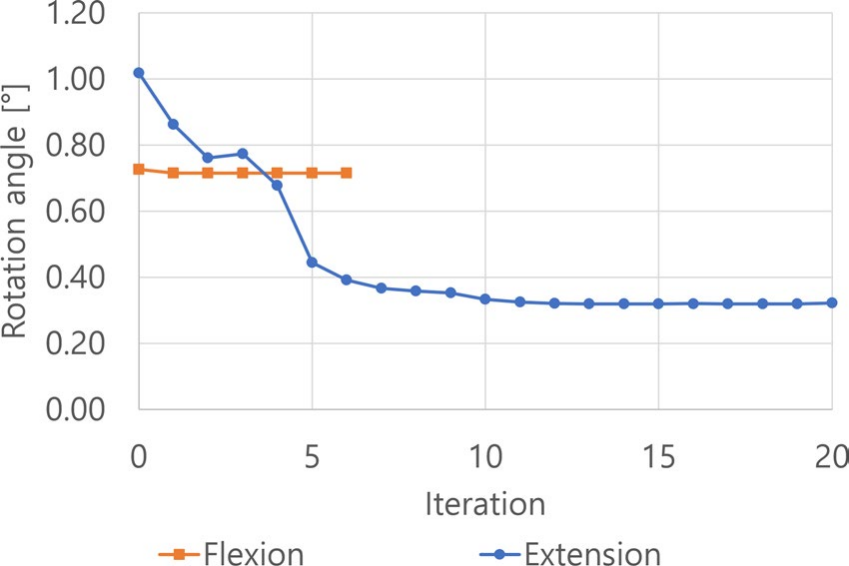
Changes in intersegmental rotation with ExGBF in flexion and extension.

The intersegmental angle slightly changed in flexion after ExGBF, whereas a distinct decrease in intersegmental rotation with ExGBF was observed in extension (Fig. 6). When extragraft bone was not formed, the intersegmental rotation angles under 1.5 Nm moments of flexion and extension were 0.73° and 1.02°, respectively. In extension, the angle decreased to 0.44° with ExGBB formation after the fifth iteration. The angle slightly changed until the ExGBF process was terminated. The final extension angle after the 20^th^ iteration was 0.32°.

### Distribution of stress and strain energy density

**Figure 7:**
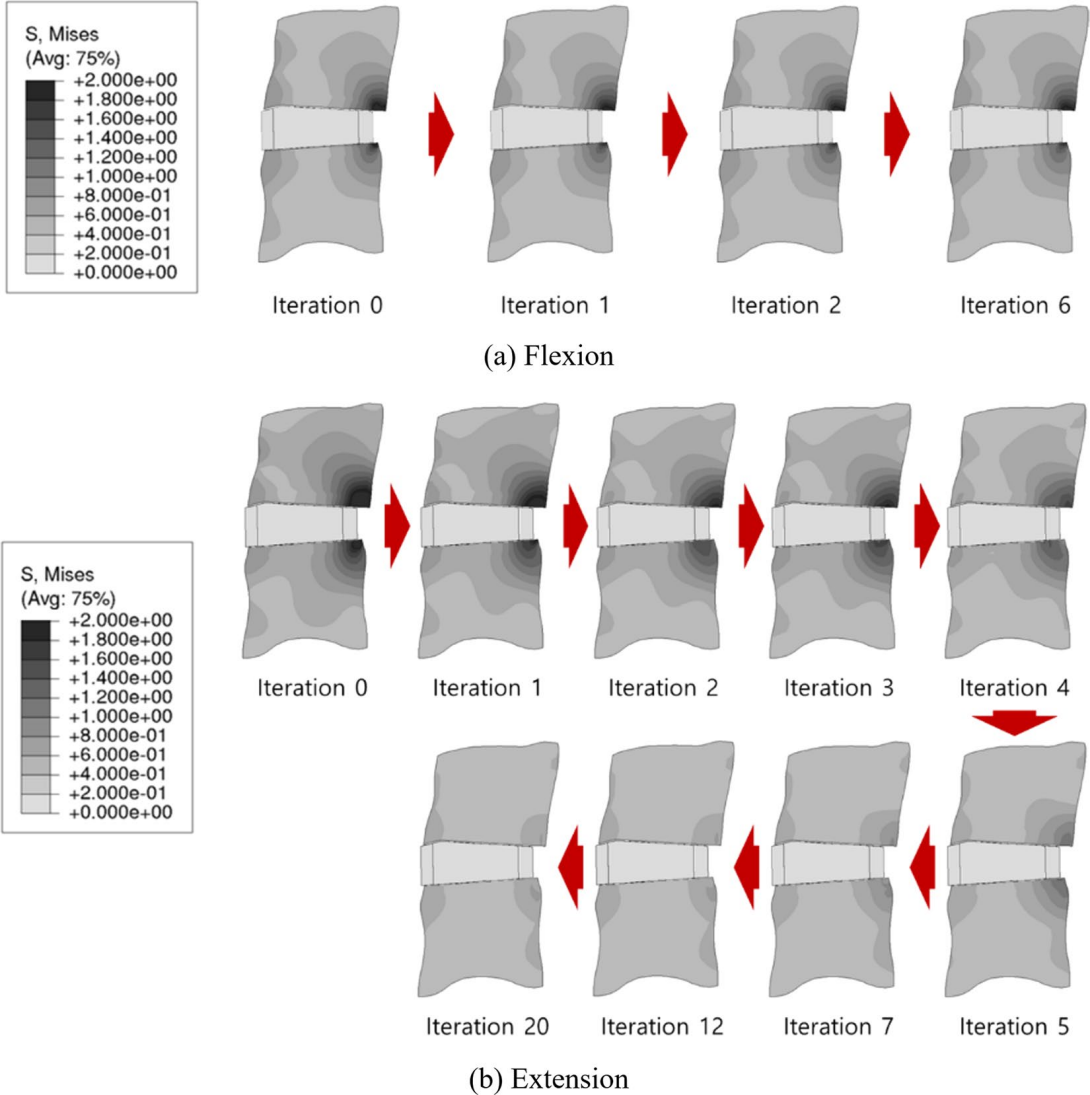
Changes in the distribution of von Mises stress on the trabecular bone with ExGBF in (**a**) flexion and (**b**) extension.

**Figure 8:**
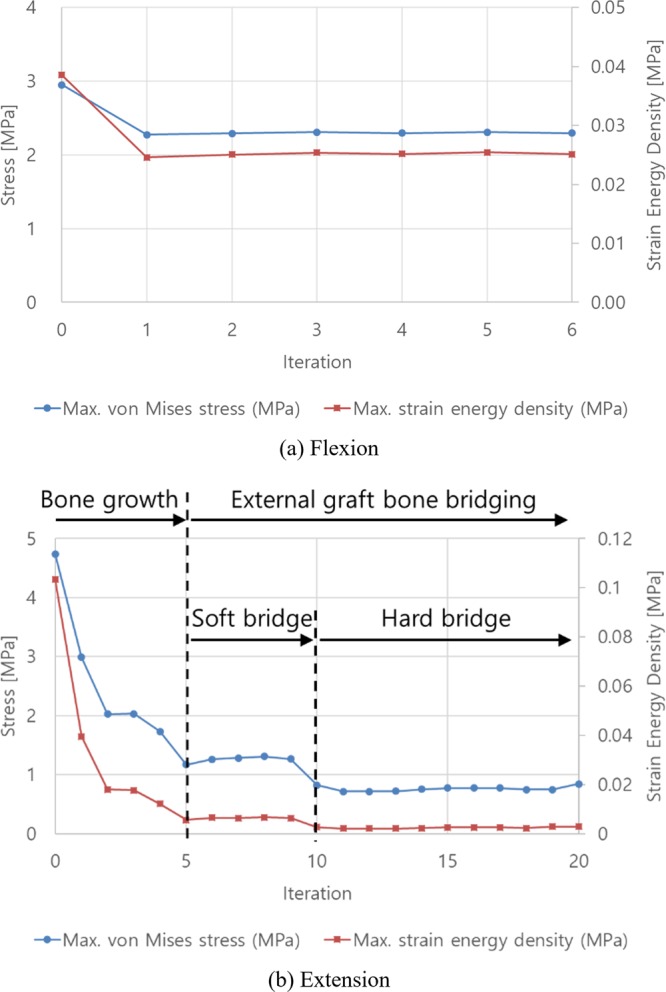
Changes in maximum von Mises stress and strain energy density with ExGBF in (**a**) flexion and (**b**) extension.

The maximum von Mises stress on the trabecular bone was predicted at the region posterior and adjacent to the cage in both flexion and extension (Fig. 7). The initial maximum von-Mises stress and SED were 2.95 MPa and 0.039 MPa in flexion, respectively, and 4.74 MPa and 0.103 MPa in extension, respectively. In flexion, both the stress and SED decreased with ExGBF and slightly changed until the bone remodelling process was terminated (Fig. 8(a)). With ExGBF, a stepwise decrease in the maximum stress and SED was predicted in extension (Fig. 8(b)). Both stress and SED decreased with the growth of ExGBF until ExGBB was formed. These values were maintained before ExGBB reached the peripheral cortical margin but decreased again as ExGBB reached the peripheral cortical margin.

## Discussion

Previously published studies have reported bone formation around inserted implants after ACDF or TDR^5,7–9^. Because formed bone can restrict intersegmental motion at the operated MSU, it is considered a risk factor in TDR^7–12^ but is essential for a solid union in ACDF. Despite its importance in both ACDF and TDR (from either positive or negative aspects, respectively), the precise cause of bone formation after surgery has not been clearly determined^7^. In this study, the authors hypothesized that bone is formed in response to the mechanical stimulus.

The segmental rotations of the developed FE model of the cervical spine were compared to the experimental results for validation. Kinematic responses of the developed models, including segmental rotation and moment-rotation curves, have been used for validation in the previously published studies^32–35^.

The kinematic responses vary according to the specimens. Thus, the experimental results showed a wide range of segmental rotation in all directions (Fig. 3). The average standard deviations of the angles under a moment of 1 Nm were 2.4°, 2.1°, 1.6°, and 1.0° in flexion, extension, left and right lateral bending, and left and right axial rotation, respectively. The predicted motions at C2-C3 and C4-C5 were not within the ranges in the experimental results in the extension and lateral bending motions, respectively. However, the differences were 0.4° in extension and 0.9° and 0.1° in the lateral bending motion. These differences are much smaller than the standard deviations of the experimental results; thus, these changes may be acceptable, considering the differences in the specimen. Although uncovertebral joints were not considered in the developed FE model in this study, a previously published finite element analysis study has shown that differences of only 4% and 6% were shown in flexion and extension motions between FE models with and without uncovertebral joints^35^. Changes in segmental rotation during flexion and extension by 4% and 6% correspond to only 0.2° and 0.3°, respectively, in the developed model. Thus, it seems that the absence of the uncovertebral joint in the developed model does not affect the validation results of the developed model.

The FE model could not simulate the neutral zone shown in the experimental results. Instead, the FE model could simulate motions with relatively lower stiffness around the neutral position using nonlinear material properties of the soft tissues, such as ligaments and intervertebral discs, affecting the kinematic response of the spinal motion segment. Nevertheless, stiffer motions were predicted in the neutral position compared with those in the experimental results. Previously published *in vitro* experimental studies have reported that the implantation of a stand-alone cage cannot eliminate the neutral zone in the lumbar and cervical spines^36–38^. Thus, the neutral zone may yield an increase in segmental rotation even at the operated motion segment of an actual spine.

The bone remodelling process leads to structural changes in the trabecular surface^39^. Wolff proposed Wolff’s law, which states that trabecular bone adapts to mechanical stimuli based on observations of the self-optimizing bone property^20^. Ganbat *et al*. estimated HO formation after cervical TDR based on Wolff’s law^18,19^. To predict the unknown geometry of HO formation, the potential region was predefined, and bone formation was simulated in the whole predefined region simultaneously. When bone adaptation is calculated simultaneously at every element in a predefined potential region connecting the upper and lower endplates, the predefined region will resist the applied load, and load sharing occurs in the region. For example, if a flexion moment is applied on a spine, a predefined potential region that is posterior to the implant, connecting the endplates, and not connecting the vertebrae cannot resist the applied load in the sequential calculations. However, a potential region connecting two vertebrae can resist the load in the simultaneous calculations. For this reason, the simultaneous calculations might overestimate the size of the HO formation. Therefore, in this study, a sequential bone remodelling algorithm was developed and used to predict ExGBF after ACDF with consideration of biological phenomena, which involve layer-by-layer bone formation by osteoclastic and osteoblastic cells, to avoid the overestimation of the size of the bone formation.

The posterior ExGBF was mainly predicted in extension. The maximum stress on the superior and inferior vertebrae was predicted in the posterior region adjacent to the cage in both flexion and extension. While compression primarily contributed to the stress in extension, tension contributed to flexion. Thus, the results explained that the growth of ExGBF is helpful in reducing the compressive stress. Extragraft bone was formed from the posterior region adjacent to the upper and lower vertebrae of the cage and subsequently grew along the posterior wall of the cage. A wedge shape was predicted before ExGBB formation. ExGBB was initially formed on the posterior residual endplate of the trabecular region and subsequently grew towards the peripheral cortical margin. The overall progress of ExGBB formation was well matched to those in medical observations (Fig. 5). These findings show that a clinician does not need to insert a cage to the posterior margin of the vertebra during ACDF and that this process does not need to be included in the pre-operative plan because ExGBF and ExGBB posterior to the inserted implant can help to stabilize the operated MSU. Moreover, the results are also helpful for physical therapists in establishing an exercise programme for a patient who underwent ACDF surgery. Although additional studies to determine the relationships between the implant position, ExGBF, and loading direction are necessary, an exercise with an extension motion may be helpful for patients whose implant location is the same as that in this study.

The stepwise decrease in the intersegmental rotation and the maximum stress and SED on the trabecular bone were predicted with ExGBF. Although the phenomenon was predicted even in cases in which the ExGBB was not formed, ExGBF showed a distinct decrease in intersegmental rotation, the maximum stress and SED. Moreover, based on the results, ExGBB can be classified as either soft or hard bridging from a biomechanical point of view. When soft bridging was formed, in which ExGBB was formed on the posterior residual endplate of the trabecular region, the stiffness of the operated MSU was approximately at the maximum value (Fig. 6). However, to minimize the stress and SED on the trabecular bone, hard bridging, in which ExGBB reached the peripheral cortical margin, is necessary (Fig. 8). Follow-up diagnoses and subsequent treatments are necessary for the success of ACDF surgery, and it is critical to understand the mechanism of ExGBF and ExGBB and its biomechanical influence on the operated MSU for diagnoses. The results of this study can be useful in identifying a current patient’s state after ACDF for a diagnosis. This finding also reveals that measurements of the segmental motion using radiographic images cannot distinguish the two phases of ExGBB because segmental rotations converge when soft bridging is formed. Therefore, precise medical images, such as CT scans, are necessary to identify the state of ExGBB. Based on the diagnosis, a surgeon can decide whether more follow-ups and subsequent treatments are necessary.

The mechanisms of both intra- and extra-bone formation have not been clearly reported, but endochondral and intramembranous ossification may be the principal causes of intra- and extragraft bone formations, respectively, in these authors’ opinion. The interbody cage surrounds the graft bone, and its Young’s modulus is much higher than that of the bone graft. Therefore, the applied load on the upper vertebra is mainly transmitted to the lower vertebra via the cage. Thus, it is not adequate to assess intragraft bone formation using the bone remodelling theory based on Wolff’s law. However, during spinal bending motions, including flexion, extension, and lateral bending motions, the stress concentration around the edge of the interbody cage can affect ExGBF.

There are several limitations in this study. The developed FE model of the cervical spine does not have a neutral zone. Moreover, the cage was constrained on the bony endplates using the 'no separation option' instead of by keels or screws to simplify the computer simulation model. These limitations must yield very small intersegmental rotations of the operated MSU within 1°. Bone remodelling occurs in the trabecular bone after ACDF, and this affects the distribution of stress and SED^17^. However, in this study, only ExGBF was considered. Although ExGBF was observed not only in the region anterior and posterior to the inserted cage but also in the lateral region, only the posterior region was chosen as a potential region, and only flexion-extension motions were considered in this study. The influences of the biological factors of patients and graft-related factors were not considered in the ExGBF and ExGBB simulations, and the algorithm was indirectly validated by comparing the predicted ExGBF with measurements on medical images because of a lack of information.

This study is the first to investigate the mechanism of ExGBF and ExGBB and its biomechanical influence on the operated MSU. Despite the abovementioned limitations, the developed sequential bone growth algorithm satisfactorily predicted the growth of ExGBF after ACDF. Therefore, the results of this study offer a rationale that supports the hypothesis of this study, which is that mechanical stimuli affect extragraft bone formation. The results of this study also provide biomechanical information on the role of extragraft bone formation and ExGBB in the operated motion segment. Extragraft bone formation was helpful in increasing the stability of the motion segment and decreasing the fracture risk of the trabecular bones. ExGBB can be classified as either soft or hard bridging from a biomechanical point of view. Numerous biomechanical factors, including the bone graft method, presence of the anterior plate, lordosis angle, bone density of the vertebra, and the location, geometry and material properties of the implant, might influence extragraft bone formation. The authors believe that the formation of ExGBB can be enhanced by controlling the biomechanical factors, and the results from this study can be applied for this purpose.
